# Anti-erosive profile of an experimental 5% SnCl₂ varnish containing different concentrations of NaF

**DOI:** 10.1590/0103-6440202203969

**Published:** 2022-03-07

**Authors:** Cristiane de Melo Alencar, Mara Eliane Soares Ribeiro, Joissi Ferrari Zaniboni, Thais Piragine Leandrin, Aryvelto Miranda Silva, Edson Alves de Campos

**Affiliations:** 1 Departamento de Dentística Restauradora, Faculdade de Odontologia de Araraquara, Universidade Estadual Paulista, UNESP - Araraquara, SP, Brazil.; 2 Laboratório de Materiais Dentários do Programa de Pós-Graduação em Odontologia, Universidade Federal do Pará, PA, Brazil.

**Keywords:** Tooth Erosion, Root dentin, Enamel, Varnish, Sodium fluoride

## Abstract

This in vitro study evaluated the anti-erosive effect of an experimental varnish containing 5% stannous chloride (SnCl₂) associated with different concentrations of NaF (NaF-free, 2.5% NaF, or 5.2% NaF) on bovine enamel and root dentin. One hundred samples were pre-eroded (0.3% citric acid, pH 2.6, 10 min) and randomized into five groups (n=10 for each substrate): Negative control - milli-Q water; NaF-free - Experimental varnish SnCl₂-free and NaF-free; 2.5 NaF - Experimental varnish 5% SnCl₂ associated with 2.5% NaF; 5.2 NaF: Experimental varnish 5% SnCl₂ associated with 5.2% NaF and positive control - Commercial varnish containing 5% NaF (Duraphat). After the varnishes were applied, the erosive and abrasive challenges were carried out for five days. Loss of tooth structure (TSL) was determined by optical profilometry, and the loss of calcium (ΔCa^2+^) using atomic absorption spectroscopy. Dentin analysis was also performed by SEM. A one-way ANOVA/Bonferroni test was performed to analyze the data (α=0.05). The experimental 2.5 NaF and 5.2 NaF groups showed greater effectiveness in preventing TSL when compared to the other groups (p <0.05), regardless of the substrate. In addition, these groups showed lower loss in Ca^2+^ content when compared to the other groups (p <0.05), for enamel and dentin. Dentin showed greater TSL and ΔCa^2+^ loss when compared to enamel in all treatments (p <0.05). The 5.2% and 2.5% NaF-containing experimental varnishes showed promising results in both, the prevention of TSL and the loss of Ca^2+^, regardless of the substrate studied.

## Resumo

Este estudo in vitro avaliou o efeito anti-erosivo de um verniz experimental contendo 5% de cloreto estanoso (SnCl₂) associado a diferentes concentrações de NaF (sem NaF, 2,5% NaF ou 5,2% NaF) sobre esmalte e dentina radicular bovinos. Cem amostras foram pré-erodidas (ácido cítrico 0,3%, pH 2,6, 10 min) e randomizadas em cinco grupos (n=10 para cada substrato): Controle negativo - água milli-Q; Sem NaF - Verniz experimental sem SnCl_2_ e sem NaF; 2,5 NaF - Verniz experimental 5% SnCl_2_ associado a 2,5% NaF; 5,2 NaF: Verniz experimental 5% SnCl_2_ associado a 5,2% NaF e controle positivo - Verniz comercial contendo 5% NaF (Duraphat). Após a aplicação dos vernizes, os desafios erosivos e abrasivos foram realizados por cinco dias. A perda de estrutura dentária (PED) foi determinada por perfilometria óptica e a perda de cálcio (ΔCa^2+)^ por espectroscopia de absorção atômica. A análise da dentina também foi realizada por MEV. Um teste ANOVA/Bonferroni de uma via foi realizado para analisar os dados (α=0,05). Os grupos experimentais 2,5 NaF e 5,2 NaF apresentaram maior eficácia na prevenção de PED quando comparados aos demais grupos (p<0,05), independentemente do substrato. Além disso, esses grupos apresentaram menor perda no teor de Ca^2+^ quando comparados aos demais grupos (p<0,05), para esmalte e dentina. A dentina apresentou maior PED e de ΔCa^2+^ quando comparada ao esmalte em todos os tratamentos (p<0,05). Os vernizes experimentais contendo NaF 5,2% e 2,5% apresentaram resultados promissores tanto na prevenção de PED quanto na perda de Ca^2+^, independente do substrato estudado.

## Introduction

During the process of dental erosion, partial demineralization of the enamel and/or dentin surface occurs due to exposure to acidic substances. However, the loss of superficial dental tissue is the result of an association between acid and mechanical challenge simultaneously or alternately ^1^. Recently, there was an international consensus on the appropriate terminology to describe the loss of dental structure through erosion and mechanical abrasion: Erosive Tooth Wear ^2^.

Frequent ingestion of acidic foods and drinks alters the structural integrity of enamel and dentin and the physical properties of these structures. This process causes the dental surface to soften along with partial loss of this altered structure. Clinically, the chemical-mechanical degradation process, enhanced by mechanical forces, accelerates erosive tooth wear ^3^ ^,^ ^4^. When the acid can diffuse through the acquired enamel pellicle, the hydrogen ions (H^+^) present in the acidic substances damage the apatite crystals present in the enamel and this starts the process of acid erosion^1^. If erosive tooth wear is continuous, it can reach the dentin and cause exposure of the dentinal tubules and dentin hypersensitivity ^5^.

Therefore, several materials and protocols have been investigated to prevent or minimize erosive dental wear on enamel ^6^ ^,^ ^7^ and dentin ^5^ ^,^ ^8^. A recent systematic review has shown that the use of stabilized stannous fluoride dentifrices can prevent the onset of tooth erosion ^9^. However, to date, no treatment has been considered the gold standard for this important issue, and the number of randomized clinical studies is very small.

Materials containing tin-like polyvalent metal ions (Sr^2+^) have previously shown some anti-erosive potential ^10^. Stannous chloride-based solutions and toothpastes (SnCl₂) have been tested previously showing promising results ^11^ ^,^ ^12^. However, SnCl₂ shows severe solubility ^13^. Stannous has a strong chemical affinity for mineralized dental tissues and promotes a protective effect due to the mechanical formation of a hypermineralized and acid-resistant surface layer ^14^. In addition, in eroded and exposed dentin, tin can be partially retained by the organic dentinal matrix ^15^.

For this reason, this study aimed to evaluate in vitro the anti-erosive potential of an experimental varnish containing 5% SnCl₂ associated with different concentrations of sodium fluoride on the enamel and dentin subjected to acid challenges. According to the authors' best knowledge, SnCl₂ has not been studied in the varnish formulation until now. The null hypotheses tested were: H01 - There is no difference in erosive dental wear between the experimental and control varnishes; H02 - There is no difference in the loss of calcium ions between the experimental and control varnishes.

## Material and Methods

### Sample preparation

This study was approved by the animal ethics committee under the identifier ID CEUA - 8031261217. Enamel and dentin blocks were obtained from 140 healthy bovine incisors using a water-cooled double-sided diamond disc (Buehler, Lake Bluff, IL, USA). The blocks with surface of enamel and root dentin (4×4×2mm^3^) were cut using an Isomet cutting machine (Buehler, Lake Bluff, Illinois, United States) with a double-sided diamond disc (Extec, Enfield, Connecticut, United States). After that, the enamel blocks were polished using silicon carbide sandpapers #600, #1200, and #2400 (3M, Sumaré, São Paulo, Brazil) and dentin blocks using silicon carbide sandpaper #600 (3M, Sumaré, São Paulo, Brazil). After polishing, the samples were immersed in an ultrasonic bath (Euronda Spa, Montecchio Precalcino, Vicenza, Italy) with distilled water (Milli-Q, Merck Millipore Corporation, Darmstadt, Germany) for 5 min ^5^. Thereafter, the samples were stored in a humid environment (Milli-Q water) at 4 °C.

### Selection of specimens

The 70 enamel and 70 dentin blocks were subjected to baseline surface microhardness (SMH. SMH was performed using Knoop microhardness (Surftest Mitsutoyo South American, São Paulo, Brazil) under a 50g load for 15 s ^16^ and 5 s ^17^, respectively. Five indentations were performed with a space of 100 µm from each other in the central area of ​​the enamel surface. After the test, data normality assessment (Shapiro - Wilk test) was performed using SPSS software version 13.0 (SPSS, Tulsa, OK, USA). Twenty dentin blocks and 20 enamel blocks were excluded, as they presented outliers microhardness values and 50 enamel and 50 dentin samples were numbered and randomized into five groups (n=10 for each substrate) using Bioestat 5.3 software (Civil Society Mamirauá, Tefé, AM, Brazil); therefore, the mean baseline microhardness values were not statistically different between groups (analysis of variance [ANOVA]; α = 0.05).

### Initial erosion

An initial erosive lesion was created by the application of citric acid at a concentration of 0.3%, pH 2.6, for 10 min. This protocol was performed on 10-well acrylic plates, and each sample was inserted into a specific well. After that, each sample was washed with distilled water for 10 s using a millimeter pick. Half of the samples' eroded surface was covered with unplasticized polyvinyl chloride (UPVC) tape to leave a 4×2 mm exposure window uncovered ^5^.

### Treatment with varnishes and erosive-abrasive challenge

After initial erosion and protection of half of the specimen's surface with UPVC tape, the varnishes were applied to the respective groups (n = 10 for each substrate): Negative control - milli-Q water; NaF-free - Experimental varnish SnCl₂-free and NaF-free; 2.5 NaF - Experimental varnish 5% SnCl₂ associated with 2.5% NaF; 5.2 NaF: Experimental varnish 5% SnCl₂ associated with 5.2% NaF and positive control - Commercial varnish containing 5% NaF (Duraphat, Colgate-Palmolive Company, Lörrach, Germany). The basic composition of the experimental varnishes incudes thickener polymer, rosin, synthetic resin, essence, and ethanol (Faculty of Pharmacy at USP Faculty of Pharmacy, São Paulo, SP, Brazil). The pH of all varnishes was measured using an indicator paper (± 0.5 units). The experimental materials showed color and consistency similar to Duraphat varnish.

**Figure 1:**
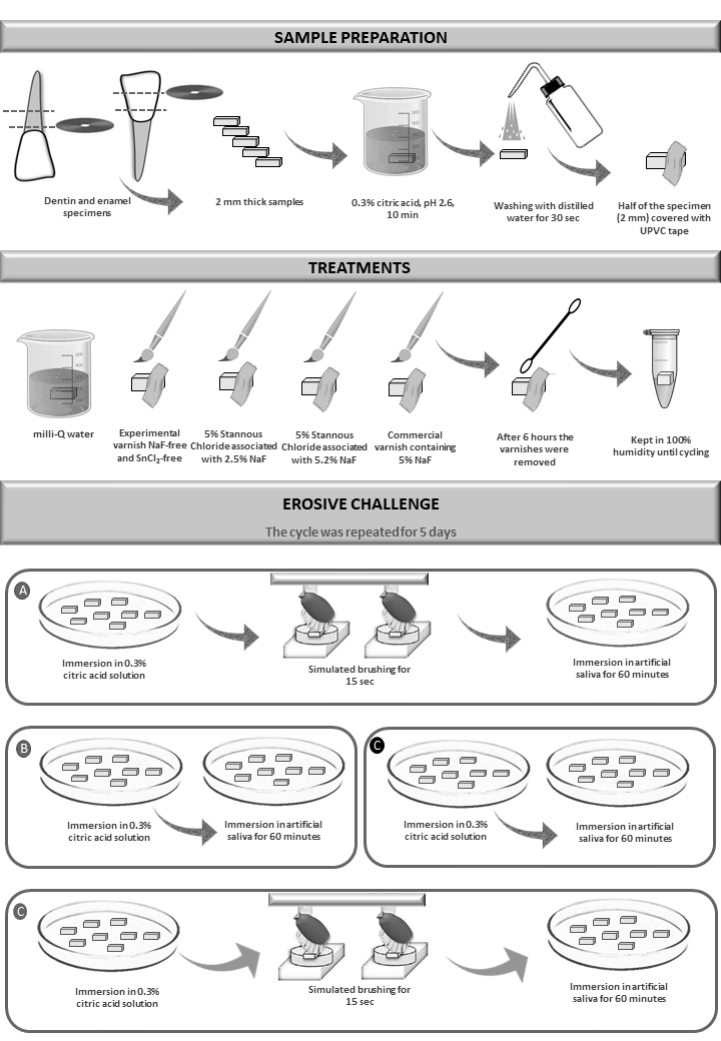
Specimen preparation, treatment and erosive-abrasive challenge. The four daily stages of the cycle were divided into: A - Erosion, abrasion and saliva immersion; B - Erosion and immersion in saliva; C - Erosion and immersion in saliva and D - Erosion, abrasion and immersion in saliva.

The varnishes were applied in a thin layer using a disposable brush and the specimens were stored in artificial saliva for 6 h. Subsequently, the varnishes were removed with acetone solution (1:1) and cotton swabs, avoiding contact with the dentin surface ^18^. In the negative control group, the specimens were immersed in milli-Q water for 6 hours. After carrying out the treatments, the samples were subjected to acid cycling for five days. The specimens of each group were immersed in a 0.3% citric acid solution (pH = 2.6 for 10 min) and then immersed in artificial saliva (Concentration of components in 0.96 g/1000 mL - KCl; NaCl; MgCl_2_; K_2_HPO_4_; CaCl_2_; Carboxymethylcellulose; Sorbitol 70%; Nipagin; Nipazole and deionized water) for 60 minutes. The samples were brushed with a simulated brushing machine (MEV-2T Odeme, Joaçaba, SC, Brazil) twice daily, calibrated in 45 cycles of, 150 g for 15 s. The simulated brushing was performed 30 min after the 1st and 4th acidic challenges on each day of the cycle. The entire protocol was performed at an average temperature of 25 °C, and at the end of each day, the samples were stored in 100% humidity (Figure 1) ^19^.

### Loss of tooth structure

The 100% humidity of the specimens was maintained throughout the experiment. The surface topography of the samples was measured by non-contact 3D profilometry (Nanovea PS50 Optical, NANOVEA, Irvine, USA). The capture was carried out with a chromatic confocal sensor with an axial source of white light, a scanning speed of 2 m/s, and a refractive index of 10,000. An area of ​​1 mm × 1 mm was obtained from the center of each sample. The analysis determined the loss of tooth structure (TSL), defined as the difference in height (Δ height) between the untreated surface (baseline) and the treated surface. The values in µm were calculated using the Nanovea Professional 3D Software and this methodology was carried out according to Alexandria, et al. ^20^.

### Loss of calcium (Ca^2+^)

The loss of Ca^*2+*^ content in the samples was calculated by subtracting the percentage of Ca^*2+*^ in mg/L (%ΔCa^2+^) content between the side treated with the materials and the eroded side (_%_ΔCa^2+^= _%_Ca^2+^ - treated - _%_Ca^2+^ - eroded). The dissolution of enamel and dentin was performed by chemical analysis on each side of the specimen. An Analyst 400 atomic absorption spectrometer (Perkin Elmer Analytical Instrument, Norwalk, CT, USA) was used with a wavelength of 422.7 nm and an air-acetylene flame was used to analyze the blind samples. 0.5% lanthanum was added to the etch samples (1:10, lanthanum: sample) to neutralize the negative effect of phosphorus on the calcium sensitivity of the spectrophotometer equipment ^21^.

### Scanning electron microscopy

The images were obtained by scanning electron microscope (TESCAN, Mira3, quanta FEG-field emission gun, Czech Republic). The samples were mounted on aluminium supports (12 mm diameter) using carbon double-sided adhesive tape and metallized with gold for 1.5 h, which deposited on the sample a film with a mean thickness of 10 to 15 nm. The images were generated by detection of secondary electrons, using voltage acceleration of 3.0 kV, working distance of around 15 mm and 2,000× magnification. The resulting micrographs were qualitatively analysed.

### Statistical analysis

SPSS software version 13.0 (SPSS, Tulsa, OK, USA) was used to perform the statistical analysis. The evaluation of the parametric distribution of data was performed using the Shapiro-Wilk test and homoscedasticity was also verified. Two-way ANOVA followed by the Bonferroni test was used. The significance level was set at α = 0.05. 

## Results

### Loss of tooth structure

The results are shown in Table 1. The experimental groups 2.5 NaF and 5.2 NaF showed lower TSL when compared to the other groups (p <0.05), for enamel and dentin. There was no statistically significant difference between groups 2.5 NaF and 5.2 NaF (Enamel: p = 0.821; Dentin: p = 0.543). The negative control group showed significantly higher TSL when compared to the other groups for both substrates (p <0.05). When comparing substrates (enamel *vs.* dentin), dentin showed TSL significantly higher than enamel in all groups (p <0.05).

**Table 1:** Mean and standard deviation (SD) of the enamel and dentin loss of tooth structure (TSL) values in µm

Group	TSL (µm)	
	Enamel	Dentin
	Mean (±SD)	Mean (±SD)
Negative control	-138.23 (±16.97)^Aa^	-321.16 (±34.37)^Ab^
NaF-free	-16.35 (±2.59)^Ba^	-42.13 (±4.09)^Bb^
2.5 NaF	-6.09 (±0.80)^Da^	-16.25 (±3.32)^Db^
5.2 NaF	-5.87 (±1.05)^Da^	-14.98 (±2.88)^Db^
Positive control	-10.86 (±1.22)^Ca^	-21.41 (±4.76)^Cb^

* Different capital letters show a statistically significant difference (p <0.05) between groups within the same substrate (Comparison between lines); Different lowercase letters show a statistically significant difference (p <0.05) between enamel and dentin substrates (comparison between columns). Note: Negative control - milli-Q water; NaF-free - Experimental varnish SnCl₂-free and NaF-free; 2.5 NaF - Experimental varnish 5% SnCl₂ associated with 2.5% NaF; 5.2 NaF: Experimental varnish 5% SnCl₂ associated with 5.2% NaF and positive control - Commercial varnish containing 5% NaF (Duraphat^®^).

### Loss of calcium (Ca^2+^)

The results of %ΔCa^2+^ are specified in Table 2. The experimental groups 2.5 NaF and 5.2 NaF showed lower loss in Ca^2+^ content when compared to the other groups (p <0.05), for enamel and dentin. There was a significant loss in Ca^2+^ content (mg/mL) in the negative control and NaF-free groups for the enamel when compared to the other groups (p <0.05). On the other hand, for dentin, the greatest loss of Ca^2+^ content occurred in the negative control group (p <0.05). When comparing substrates (enamel vs. dentin), enamel showed TSL significantly higher when compared to dentin in all groups (p <0.05).

**Table 2:** Mean and standard deviation (SD) of the enamel and dentin loss of calcium (ΔCa^2+^) values in % (mg/mL)

Group	_%_ **ΔCa** ^2+^ **(mg/mL)**	
	Enamel	Dentin
	Mean (±SD)	Mean (±SD)
Negative control	-1.52 (±0.54)^Aa^	-0.57 (±0.12)^Ab^
NaF-free	-0.82 (±0.14)^Ba^	-0.18 (±0.05)^Bb^
2.5 NaF	-0.14 (±0.07)^Da^	-0.06 (±0.03)^Cb^
5.2 NaF	-0.11 (±0.04)^Da^	-0.04 (±0.02)^Cb^
Duraphat	-0.36 (±0.13)^Ca^	-0.16 (±0.06)^Bb^

* Different capital letters show a statistically significant difference (p <0.05) between groups within the same substrate (Comparison between lines); Different lowercase letters show a statistically significant difference (p <0.05) between enamel and dentin substrates (comparison between columns). Note: Negative control - milli-Q water; NaF-free - Experimental varnish SnCl₂-free and NaF-free; 2.5 NaF - Experimental varnish 5% SnCl₂ associated with 2.5% NaF; 5.2 NaF: Experimental varnish 5% SnCl₂ associated with 5.2% NaF and positive control - Commercial varnish containing 5% NaF (Duraphat^®^).

### Scanning electron microscopy

**Figure 2:**
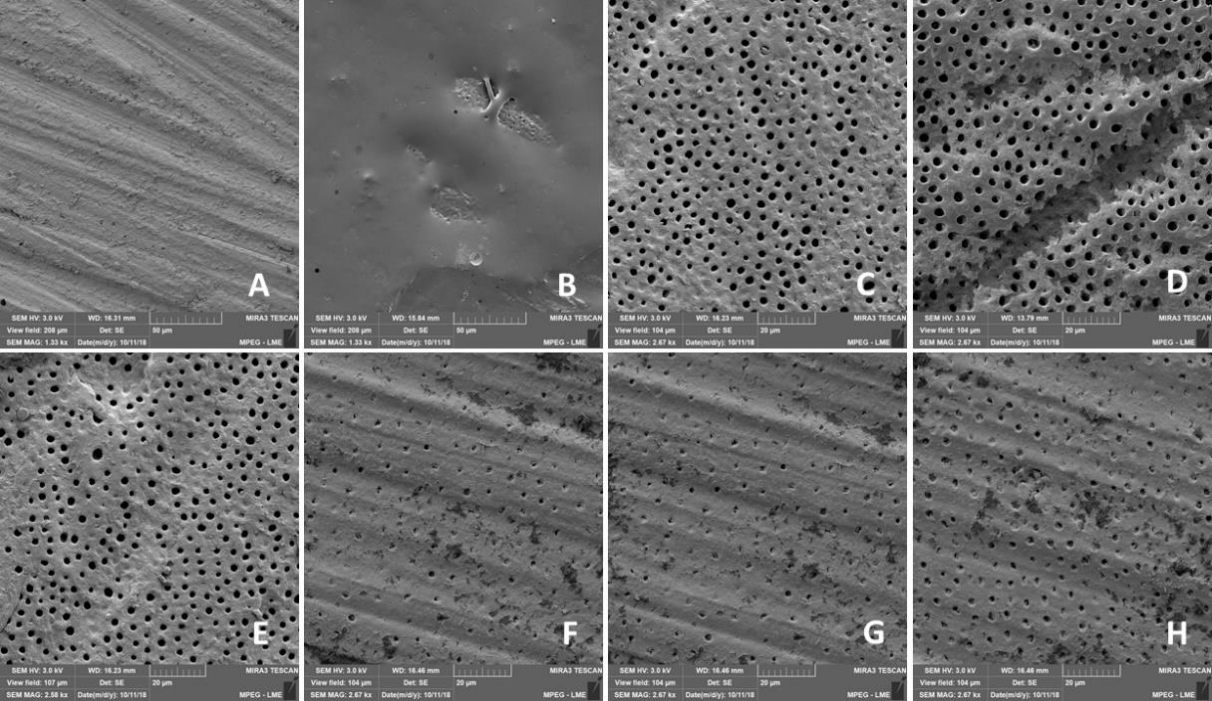
A - Dentin with smear layer; B - Dentin after applying the varnish; C - Dentin after initial erosion; D - Negative control; E - NaF-free; F - 2.5 NaF; G - 5.2 NaF and H - positive control

Scanning electron microscopy images are shown in Figure 2. Analysis of the samples after flattening with a #600-grit abrasive disc (Figure 2A) showed a total obliteration pattern of the dentin tubules due to the presence of the smear layer. Figure 2B illustrates the dentin surface covered by the experimental varnish containing 5.2% NaF, before being removed. After the erosion protocol with 0.3% citric acid (Figure 2C), the dentin surface revealed notable open dentinal tubules with a larger diameter. 

The qualitative surface analysis showed that, among the groups studied, only 2.5 NaF (Figure 2F), 5.2 NaF (Figure 2G) and positive control (Figure 2H) showed partial obliteration of dentinal tubules. 

## Discussion

The current concern with ETW is growing^3^; therefore, several preventive treatments have been investigated. Among these therapies, toothpastes with and without NaF ^5^ ^,^ ^21^ ^,^ ^22^, varnishes ^16^ ^,^ ^20^, and bioactive materials ^24^ ^,^ ^25^ have shown promising results. However, the literature presents conflicting results and few randomized clinical studies on this issue. In the present in vitro research, the anti-erosive potential of two experimental varnishes was tested and showed positive results in preventing ETW in enamel and dentin. For this reason, hypothesis H01 was rejected.

Stannous (Sn^2+^) is a polyvalent metal ion that has a strong affinity for mineralized dental tissues. Sn^2+^ promotes a protective effect on dental tissue, acting on the formation of an acid-resistant surface layer ^26^. A previous study showed that SnCl_2_/NaF in solution form was able to prevent enamel and dentin erosion for 6 and 3.5 minutes, respectively ^26^. The most pronounced protective effect at the beginning of the acid challenge may be related to the incorporation of stannous in the outermost enamel layer ^27^. An important study conducted by Babcock et al. ^28^ showed that the anti-erosive effect of the combination between Sn^2+^ and F^-^ occurs due to formation of less soluble precipitates on dental surface. Another study by Ganss, et al. ^29^ showed that the anti-erosive potential of NaF was better than SnCl_2_, but less than its combination.

In the present study, experimental varnishes based on SnCl_2_ associated with NaF showed promising results in decreasing TSL in enamel and dentin, regardless of NaF concentration. In this sense, it can be assumed that, in the present investigation, the combination of Sn^2+^ and F^-^ were incorporated into the structure of enamel and dentin, improving its acid resistance ^30^. In addition, varnishes are materials that require less clinical applications compared to toothpastes and solutions because their effects last longer ^5^. A study by Sancakli, et al. ^(^ ^31^ showed that topical fluoride varnish treatments have a superficial and sub-superficial effect, which plays a significant role in the prevention of TSL. There was no difference between the varnishes with a concentration of 2.5% and 5.2% NaF in the prevention of TSL during the erosive challenge in this study. It is possible that the effect of 5.2% NaF on TSL saturates the NaF concentration effectiveness threshold. Thus, the 2.5% concentration has already proved to be sufficiently efficient for an anti-erosion effect.^^

There are morphological and structural differences reported in the literature between dentin and enamel substrates ^32^. In the present study, we used the bovine buccal enamel and the root dentin of the cervical portion, which is predominantly involved in cervical lesions that are not carious by erosion ^5^ ^,^ ^33^. Bovine dentin and enamel demonstrate a structural biomorphology similar to the human substrate, including the quantity and density of dentinal tubules and a similar collagen matrix ^34^. It was previously concluded that the use of bovine teeth in vitro is acceptable, mainly for comparisons of effectiveness between materials ^35^; justifying the use of this substrate in this research.

In the present study, it was observed that dentin had a higher TSL than enamel. In contrast, the dentin structure showed lower loss of Ca^2+^ when compared to enamel. Thus, H02 was rejected. This can be explained by the differences between these substrates ^32^, considering that the enamel has a higher inorganic content and consists of solid, interlaced, and rod-shaped structures. There is a large concentration of metal ions such as Na^+^, K^+^, and Mg^2+^ inside the enamel, while ions F^-^ and Cl^-^ are more prevalent on its surface ^32^ ^,^ ^36^. On the other hand, dentin is a less mineralized substrate, thus justifying the higher TSL compared to enamel ^37^. Previous studies have shown a potential protective effect of solutions containing F^-^ and Sn^2+^ on TSL in the enamel ^10^ ^,^ ^38^ ^,^ ^39^. This was attributed to the formation of a layer of poorly soluble precipitates, with Sn_2_OHPO_4_, Sn_3_F_3_PO_4_, and Ca(SnF_3_)_2_ on the enamel surface ^28^. In addition, another study showed that Sn^2+^ can be incorporated into the enamel structure ^39^ ^,^ ^40^.

The experimental varnishes containing 2.5% and 5.2% NaF and the commercial varnish Duraphat promoted a greater pattern of dentinal tubule obliteration when compared to the negative control and NaF-free groups. Although the Duraphar varnish did not show promising results in TSL and Ca^2+^ loss, it was able to partially obliterate the dentin tubules. These results are possibly due to the remineralizing potential of NaF in these groups. The Duraphat varnish has a high concentration of sodium fluoride (22,600 ppm) and its effect is attributed to the formation of a CaF_2_ layer on the surface, occluding the dentinal tubules. 

The role of saliva is fundamental during the erosive challenge on dental tissues, as it acts in the formation of the acquired film. This film membrane is a semi-permeable structure that can decrease the contact of acids with the dental tissue ^41^. The performance of the acquired film in the face of acid challenges must be considered in methodologies involving erosion and/or abrasion cycles. In the present study, we did not measure the thickness of the acquired film formed or the degree of its interference in the results. It is possible that this is a limitation of this study. However, it is important to consider that all groups were subjected to the same erosion/abrasion conditions.

Although in vitro studies aim to faithfully mimic the oral cavity in a controlled environment, there are variables that were not considered in this study: change in intraoral temperature, masticatory forces, and changes in salivary flow, etc. Further randomized controlled clinical studies with a low risk of bias should be conducted to evaluate promising alternatives for the prevention of TSL. However, testing of experimental materials must first be carried out in vitro and, if it shows promising results, it can be tested clinically in the future. The experimental varnishes tested in this research should be perfected in future in vitro research before they are suitable for clinical application.

Within the limitations of the present study, we can conclude that the 5.2% and 2.5% NaF-containing experimental varnishes showed promising results, both in the prevention of erosive tooth loss and loss of Ca^2+^, regardless of the substrate. In addition, dentin showed greater erosive tooth loss and less Ca^2+^ loss when compared to enamel in all treatments.
